# *N*-[(2*S*)-4-Chloro-2-(l-menth­yloxy)-5-oxo-2,5-dihydro­furan-3-yl]-l-valine

**DOI:** 10.1107/S1600536809022120

**Published:** 2009-06-17

**Authors:** Xiu-Mei Song, Zhao-Yang Li, Zhao-Yang Wang, Jian-Xiao Li

**Affiliations:** a School of Chemistry and Environment, South China Normal University, Guangzhou 510006, People’s Republic of China

## Abstract

The title compound, C_19_H_30_ClNO_5_, was obtained by the tandem asymmetric Michael addition–elimination reaction of (5*S*)-3,4-dichloro-5-(l-menth­yloxy)furan-2(5*H*)-one and l-valine in the presence of potassium hydroxide. The furan­one unit is approximately planar (r.m.s. deviation = 0.0204 Å) and the six-membered cyclo­hexane ring adopts a chair conformation. The crystal structure is stabilized by a network of O—H⋯O and N—H⋯O hydrogen bonds.

## Related literature

For biologically active 4-amino-2(5*H*)-furan­ones, see: Kimura *et al.* (2000 ▶); Tanoury *et al.*, 2008 ▶). For the synthesis of the precursor, (5*S*)-3,4-dichloro-5-(l-menth­yloxy)furan-2(5*H*)-one, see: Chen & Geng (1993 ▶). 
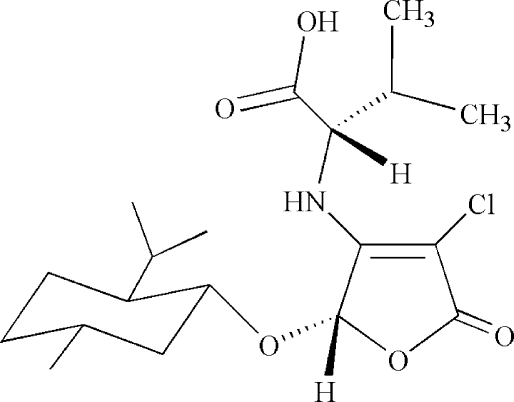


## Experimental

### 

#### Crystal data

C_19_H_30_ClNO_5_
*M* *_r_* = 387.89
Tetragonal, 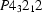

*a* = 10.4540 (4) Å
*c* = 39.300 (3) Å
*V* = 4294.9 (4) Å^3^
*Z* = 8
Mo *K*α radiation
μ = 0.20 mm^−1^
*T* = 293 K
0.30 × 0.23 × 0.15 mm

#### Data collection

Bruker APEXII CCD area-detector diffractometer
Absorption correction: multi-scan (*SADABS*; Bruker, 2004 ▶) *T*_min_ = 0.769, *T*_max_ = 0.867 (expected range = 0.860–0.970)
22031 measured reflections
3796 independent reflections
2868 reflections with *I* > 2σ(*I*)
*R*_int_ = 0.053

#### Refinement

*R*[*F* ^2^ > 2σ(*F* ^2^)] = 0.040
*wR*(*F* ^2^) = 0.091
*S* = 1.04
3796 reflections
242 parameters
H-atom parameters constrained
Δρ_max_ = 0.18 e Å^−3^
Δρ_min_ = −0.23 e Å^−3^
Absolute structure: Flack, (1983 ▶), 1499 Friedel pairs
Flack parameter: −0.03 (8)

### 

Data collection: *APEX2* (Bruker, 2004 ▶); cell refinement: *APEX2* and *SAINT* (Bruker, 2004 ▶); data reduction: *SAINT*; program(s) used to solve structure: *SHELXS97* (Sheldrick, 2008 ▶); program(s) used to refine structure: *SHELXL97* (Sheldrick, 2008 ▶); molecular graphics: *SHELXTL* (Sheldrick, 2008 ▶); software used to prepare material for publication: *SHELXL97*.

## Supplementary Material

Crystal structure: contains datablocks I, global. DOI: 10.1107/S1600536809022120/gk2204sup1.cif

Structure factors: contains datablocks I. DOI: 10.1107/S1600536809022120/gk2204Isup2.hkl

Additional supplementary materials:  crystallographic information; 3D view; checkCIF report

## Figures and Tables

**Table 1:** Hydrogen-bond geometry (Å, °)

*D*—H⋯*A*	*D*—H	H⋯*A*	*D*⋯*A*	*D*—H⋯*A*
N1—H1⋯O2^i^	0.86	2.25	3.019 (3)	148
O1—H1*A*⋯O3^ii^	0.82	1.83	2.617 (2)	160

Symmetry codes: (i) 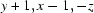; (ii) 
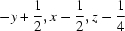
.

**Fig. 1.**
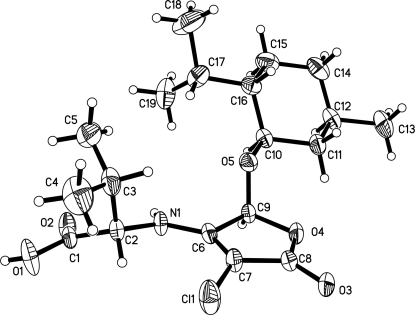
Molecular structure of the title compound with displacement ellipsoids shown at the 30% probability level.

**Fig. 2.**
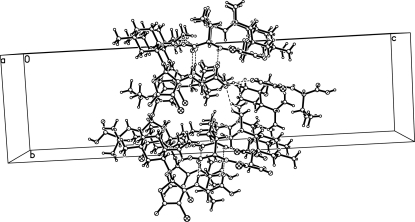
Perspective view of the crystal packing. Dashed lines represent hydrogen bonds.

## supplementary crystallographic information

### Comment

Many 4-amino-2(5*H*)-furanones have been patented as prodrugs or insecticides and herbicides (Kimura *et al.*, 2000; Tanoury *et al.*, 2008). Attracted by versatility of 4-amino-2(5*H*)-furanones, we synthesized the title molecule with chiral synthon 3,4-dichloro-5-(*S*)-(*l*-menthyloxy)-2(5*H*)-furanone and L-valine in the presence of potassium hydroxide *via* the tandem asymmetric Michael addition-elimination reaction. With 2(5*H*)-furanone moiety and polyfunctional groups (carboxyl, amino, halogeno), the title compound is expected to be a biologically active product and an excellent ligand.

The structure of the title compound is illustrated in Fig. 1. The five-membered furane ring and the six-membered cyclohexane ring are connected *via* C10—O2—C11 ether bond. The configuration of chiral centers is following: C4(*S*), C10(*S*), C11(*R*), C12(*S*), C17(*R*)). The furanone unit is approximately planar, whereas the cyclohexane ring shows a chair conformation with three substituents occupying equatorial positions. The molecules are linked by O4—H6···O3 and N1—H1···O5 hydrogen bonds forming a three-dimensional network (Table. 1 and Fig. 2).

### Experimental

The precursor, 3,4-dichloro-5-(*S*)-(L-menthyloxy)-2(5*H*)-furanone, was prepared according to the literature procedure (Chen *et al.*, 1993).

After the mixture of L-valine (4.5 mmol) and potassium hydroxide (5.8 mmol) was dissolved in absolute ethyl alcohol under nitrogen atmosphere, dichloromethane solution of 3,4-dichloro-5-(*S*)-(*l*-menthyloxy)-2(5*H*)-furanone (3.0 mmol) was added. The reaction was carried out under the stirring at room temperature for 24 h. Once the reaction was complete, the solvents were removed under reduced pressure. The residual solid was dissolved in dichloromethane, and pH of the solution was adjusted to 3–4 with 15% of aqueous HCl solution. Then the combined organic layers from extraction were concentrated under reduced pressure, and the crude product was purified by silica gel column chromatography with the gradient mixture of petroleum ether and ethyl acetate to give the product yielding (I) 0.6891 g (59.2%). Data for (I): [α]^20°^_D_ = 47.616° (c 0.481, CH_3_CH_2_OH); ^1^H NMR (400 MHz, CDCl_3_, TMS): 0.832 (3*H*, *d*, *J* = 6.0 Hz, CH_3_), 0.904–0.935 (7*H*, *m*, CH, 2CH_3_), 0.955–1.057 (8*H*, *m*, 2CH_3_, CH_2_), 1.312–1.457 (2*H*, *m*, 2CH), 1.605–1.710 (2*H*, *m*, CH_2_), 2.100–2.350 (3*H*, *m*, CH_2_, CH), 3.505–3.609 (1*H*, *m*, CH), 4.726 (1*H*, *s*, NH), 5.116–5.138 (1*H*, *d*, *J* = 8.8 Hz, CH), 5.700 (1*H*, *s*, CH), 10.212 (1*H*, *s*, COOH); ESI-MS, *m*/*z* (%): Calcd for C_19_H_31_ClNO_5_^+^([*M*+H]^+^): 388.19, Found: 388.15 (100.0)

### Refinement

All H atoms were positioned in calculated positions (O—H = 0.82 Å; N—H = 0.86 Å; C—H = 0.96Å - 0.98 Å) and were refined using a riding model, with *U*_iso_(H) = 1.2 *U*_eq_(C,N) for methylene, methine and amino H atoms and *U*_iso_(H) = 1.5 *U*_eq_(C,O) for methyl or hydroxyl H atoms.

### Crystal data

**Table:**

C_19_H_30_ClNO_5_	*D*_x_ = 1.200 Mg m^−^^3^
*M**_r_* = 387.89	Mo *K*α radiation, λ = 0.71073 Å
Tetragonal, *P*4_3_2_1_2	Cell parameters from 3405 reflections
Hall symbol: P 4nw 2abw	θ = 2.2–19.1°
*a* = 10.4540 (4) Å	µ = 0.20 mm^−^^1^
*c* = 39.300 (3) Å	*T* = 293 K
*V* = 4294.9 (4) Å^3^	Block, colourless
*Z* = 8	0.30 × 0.23 × 0.15 mm
*F*(000) = 1664.0	

### Data collection

**Table:**

Bruker APEXII CCD area-detector diffractometer	3796 independent reflections
Radiation source: fine-focus sealed tube	2868 reflections with *I* > 2σ(*I*)
graphite	*R*_int_ = 0.053
Detector resolution: 0 pixels mm^-1^	θ_max_ = 25.0°, θ_min_ = 2.0°
φ and ω scans	*h* = −12→10
Absorption correction: multi-scan (*SADABS*; Bruker, 2004)	*k* = −11→12
*T*_min_ = 0.769, *T*_max_ = 0.867	*l* = −46→46
22031 measured reflections	

### Refinement

**Table:**

Refinement on *F*^2^	Hydrogen site location: inferred from neighbouring sites
Least-squares matrix: full	H-atom parameters constrained
*R*[*F*^2^ > 2σ(*F*^2^)] = 0.040	*w* = 1/[σ^2^(*F*_o_^2^) + (0.0329*P*)^2^ + 0.7758*P*] where *P* = (*F*_o_^2^ + 2*F*_c_^2^)/3
*wR*(*F*^2^) = 0.091	(Δ/σ)_max_ = 0.001
*S* = 1.04	Δρ_max_ = 0.18 e Å^−^^3^
3796 reflections	Δρ_min_ = −0.23 e Å^−^^3^
242 parameters	Extinction correction: *SHELXL97* (Sheldrick, 2008), Fc^*^=kFc[1+0.001xFc^2^λ^3^/sin(2θ)]^-1/4^
0 restraints	Extinction coefficient: 0.0020 (3)
Primary atom site location: structure-invariant direct methods	Absolute structure: Flack, (1983), 1499 Friedel pairs
Secondary atom site location: difference Fourier map	Flack parameter: −0.03 (8)

### Special details

**Table:**

Geometry. All e.s.d.'s (except the e.s.d. in the dihedral angle between two l.s. planes) are estimated using the full covariance matrix. The cell e.s.d.'s are taken into account individually in the estimation of e.s.d.'s in distances, angles and torsion angles; correlations between e.s.d.'s in cell parameters are only used when they are defined by crystal symmetry. An approximate (isotropic) treatment of cell e.s.d.'s is used for estimating e.s.d.'s involving l.s. planes.
Refinement. Refinement of *F*^2^ against ALL reflections. The weighted *R*-factor *wR* and goodness of fit *S* are based on *F*^2^, conventional *R*-factors *R* are based on *F*, with *F* set to zero for negative *F*^2^. The threshold expression of *F*^2^ > σ(*F*^2^) is used only for calculating *R*-factors(gt) *etc*. and is not relevant to the choice of reflections for refinement. *R*-factors based on *F*^2^ are statistically about twice as large as those based on *F*, and *R*- factors based on ALL data will be even larger.

### Fractional atomic coordinates and isotropic or equivalent isotropic displacement parameters (Å^2^)

**Table:**

	*x*	*y*	*z*	*U*_iso_*/*U*_eq_	
C1	0.6886 (3)	−0.0142 (2)	−0.02302 (5)	0.0457 (6)	
C2	0.6349 (2)	0.0184 (2)	0.01161 (5)	0.0434 (6)	
H2	0.5486	−0.0178	0.0132	0.052*	
C3	0.6252 (3)	0.1638 (3)	0.01705 (6)	0.0645 (8)	
H3	0.6169	0.1771	0.0416	0.077*	
C4	0.5046 (4)	0.2198 (3)	0.00080 (9)	0.1066 (14)	
H4A	0.4929	0.3061	0.0085	0.160*	
H4B	0.4318	0.1692	0.0072	0.160*	
H4C	0.5135	0.2191	−0.0235	0.160*	
C5	0.7443 (4)	0.2337 (3)	0.00618 (8)	0.0901 (11)	
H5A	0.8180	0.1926	0.0158	0.135*	
H5B	0.7403	0.3207	0.0140	0.135*	
H5C	0.7507	0.2325	−0.0182	0.135*	
C6	0.6726 (2)	−0.0701 (2)	0.06900 (5)	0.0420 (6)	
C7	0.5557 (2)	−0.0781 (2)	0.08334 (5)	0.0478 (6)	
C8	0.5677 (3)	−0.1196 (2)	0.11793 (5)	0.0489 (6)	
C9	0.7698 (2)	−0.0992 (2)	0.09642 (5)	0.0429 (6)	
H9	0.8263	−0.1695	0.0896	0.052*	
C10	0.9662 (2)	−0.0054 (2)	0.11656 (5)	0.0445 (6)	
H10	1.0136	−0.0663	0.1024	0.053*	
C11	0.9624 (2)	−0.0546 (3)	0.15302 (5)	0.0529 (7)	
H11A	0.9221	−0.1381	0.1534	0.063*	
H11B	0.9112	0.0029	0.1668	0.063*	
C12	1.0967 (3)	−0.0645 (3)	0.16813 (6)	0.0596 (7)	
H12	1.1447	−0.1278	0.1549	0.072*	
C13	1.0913 (3)	−0.1106 (3)	0.20518 (6)	0.0868 (10)	
H13A	1.0524	−0.0458	0.2191	0.130*	
H13B	1.1764	−0.1271	0.2132	0.130*	
H13C	1.0416	−0.1877	0.2065	0.130*	
C14	1.1649 (3)	0.0631 (3)	0.16501 (7)	0.0753 (9)	
H14A	1.1233	0.1250	0.1797	0.090*	
H14B	1.2525	0.0534	0.1727	0.090*	
C15	1.1654 (3)	0.1133 (3)	0.12899 (7)	0.0723 (9)	
H15A	1.2151	0.0562	0.1147	0.087*	
H15B	1.2062	0.1966	0.1286	0.087*	
C16	1.0302 (2)	0.1249 (2)	0.11446 (6)	0.0522 (7)	
H16	0.9828	0.1814	0.1299	0.063*	
C17	1.0229 (3)	0.1862 (3)	0.07910 (7)	0.0698 (8)	
H17	0.9327	0.1846	0.0723	0.084*	
C18	1.0627 (4)	0.3269 (3)	0.08046 (11)	0.1295 (16)	
H18A	1.0213	0.3678	0.0993	0.194*	
H18B	1.0380	0.3685	0.0597	0.194*	
H18C	1.1538	0.3325	0.0832	0.194*	
C19	1.0969 (4)	0.1145 (4)	0.05192 (7)	0.1001 (12)	
H19A	1.1866	0.1177	0.0571	0.150*	
H19B	1.0818	0.1534	0.0302	0.150*	
H19C	1.0692	0.0270	0.0514	0.150*	
Cl1	0.40649 (7)	−0.05720 (10)	0.066293 (16)	0.0818 (3)	
N1	0.71379 (18)	−0.0411 (2)	0.03775 (4)	0.0463 (5)	
H1	0.7919	−0.0586	0.0327	0.056*	
O1	0.60255 (18)	0.0096 (2)	−0.04637 (4)	0.0835 (7)	
H1A	0.6322	−0.0068	−0.0652	0.125*	
O2	0.79397 (18)	−0.05167 (19)	−0.02856 (4)	0.0633 (5)	
O3	0.48370 (18)	−0.14530 (18)	0.13838 (4)	0.0625 (5)	
O4	0.69263 (17)	−0.13404 (16)	0.12582 (3)	0.0515 (5)	
O5	0.83805 (15)	0.01070 (15)	0.10286 (4)	0.0472 (4)	

### Atomic displacement parameters (Å^2^)

**Table:**

	*U*^11^	*U*^22^	*U*^33^	*U*^12^	*U*^13^	*U*^23^
C1	0.0580 (17)	0.0518 (16)	0.0274 (11)	0.0057 (12)	−0.0053 (11)	0.0020 (11)
C2	0.0501 (15)	0.0544 (16)	0.0259 (10)	0.0075 (12)	−0.0023 (10)	0.0030 (10)
C3	0.095 (2)	0.0608 (18)	0.0382 (13)	0.0207 (17)	0.0001 (14)	−0.0027 (13)
C4	0.135 (4)	0.086 (3)	0.099 (2)	0.056 (2)	−0.020 (2)	0.002 (2)
C5	0.132 (3)	0.067 (2)	0.072 (2)	−0.014 (2)	0.006 (2)	0.0021 (17)
C6	0.0515 (15)	0.0469 (15)	0.0276 (11)	0.0046 (11)	−0.0012 (11)	−0.0033 (10)
C7	0.0480 (15)	0.0655 (17)	0.0301 (11)	0.0041 (13)	0.0008 (11)	0.0020 (11)
C8	0.0632 (19)	0.0531 (16)	0.0303 (11)	−0.0084 (13)	0.0000 (12)	−0.0034 (11)
C9	0.0542 (15)	0.0481 (15)	0.0265 (11)	−0.0019 (12)	−0.0016 (10)	0.0020 (11)
C10	0.0485 (15)	0.0488 (15)	0.0361 (12)	0.0063 (12)	−0.0051 (11)	−0.0040 (11)
C11	0.0620 (17)	0.0601 (17)	0.0365 (12)	0.0011 (14)	−0.0035 (12)	−0.0009 (12)
C12	0.0670 (18)	0.070 (2)	0.0418 (13)	0.0108 (15)	−0.0119 (13)	−0.0125 (13)
C13	0.106 (3)	0.108 (3)	0.0464 (16)	0.017 (2)	−0.0248 (17)	−0.0055 (17)
C14	0.070 (2)	0.092 (3)	0.0641 (18)	−0.0008 (18)	−0.0196 (15)	−0.0204 (17)
C15	0.064 (2)	0.073 (2)	0.079 (2)	−0.0123 (17)	−0.0053 (16)	−0.0122 (17)
C16	0.0554 (17)	0.0466 (16)	0.0545 (15)	0.0019 (12)	0.0008 (13)	−0.0082 (12)
C17	0.074 (2)	0.0622 (19)	0.0737 (19)	−0.0058 (16)	0.0041 (16)	0.0165 (15)
C18	0.153 (4)	0.071 (3)	0.164 (4)	−0.030 (3)	0.003 (3)	0.035 (3)
C19	0.119 (3)	0.123 (3)	0.0585 (18)	0.011 (3)	0.014 (2)	0.019 (2)
Cl1	0.0488 (4)	0.1483 (8)	0.0483 (4)	0.0136 (5)	0.0026 (3)	0.0132 (4)
N1	0.0460 (12)	0.0683 (14)	0.0247 (9)	0.0099 (10)	0.0023 (8)	0.0061 (9)
O1	0.0698 (13)	0.151 (2)	0.0296 (8)	0.0369 (14)	−0.0086 (9)	−0.0045 (11)
O2	0.0639 (13)	0.0890 (15)	0.0369 (9)	0.0293 (11)	0.0062 (8)	0.0049 (9)
O3	0.0717 (13)	0.0841 (14)	0.0317 (8)	−0.0182 (10)	0.0114 (9)	0.0017 (8)
O4	0.0601 (12)	0.0674 (12)	0.0270 (8)	−0.0094 (9)	−0.0030 (8)	0.0084 (7)
O5	0.0530 (11)	0.0463 (11)	0.0422 (9)	0.0000 (8)	−0.0070 (8)	0.0009 (8)

### Geometric parameters (Å, °)

**Table:**

C1—O2	1.189 (3)	C11—C12	1.528 (3)
C1—O1	1.309 (3)	C11—H11A	0.9700
C1—C2	1.511 (3)	C11—H11B	0.9700
C2—N1	1.456 (3)	C12—C14	1.517 (4)
C2—C3	1.538 (3)	C12—C13	1.534 (3)
C2—H2	0.9800	C12—H12	0.9800
C3—C5	1.506 (4)	C13—H13A	0.9600
C3—C4	1.530 (4)	C13—H13B	0.9600
C3—H3	0.9800	C13—H13C	0.9600
C4—H4A	0.9600	C14—C15	1.510 (4)
C4—H4B	0.9600	C14—H14A	0.9700
C4—H4C	0.9600	C14—H14B	0.9700
C5—H5A	0.9600	C15—C16	1.529 (4)
C5—H5B	0.9600	C15—H15A	0.9700
C5—H5C	0.9600	C15—H15B	0.9700
C6—N1	1.336 (3)	C16—C17	1.532 (3)
C6—C7	1.348 (3)	C16—H16	0.9800
C6—C9	1.511 (3)	C17—C19	1.518 (4)
C7—C8	1.432 (3)	C17—C18	1.529 (4)
C7—Cl1	1.712 (2)	C17—H17	0.9800
C8—O3	1.220 (3)	C18—H18A	0.9600
C8—O4	1.351 (3)	C18—H18B	0.9600
C9—O5	1.376 (3)	C18—H18C	0.9600
C9—O4	1.455 (3)	C19—H19A	0.9600
C9—H9	0.9800	C19—H19B	0.9600
C10—O5	1.454 (3)	C19—H19C	0.9600
C10—C16	1.520 (3)	N1—H1	0.8600
C10—C11	1.523 (3)	O1—H1A	0.8200
C10—H10	0.9800		
			
O2—C1—O1	124.8 (2)	C14—C12—C11	109.9 (2)
O2—C1—C2	125.7 (2)	C14—C12—C13	111.7 (2)
O1—C1—C2	109.5 (2)	C11—C12—C13	110.9 (2)
N1—C2—C1	109.21 (18)	C14—C12—H12	108.1
N1—C2—C3	111.14 (19)	C11—C12—H12	108.1
C1—C2—C3	111.9 (2)	C13—C12—H12	108.1
N1—C2—H2	108.2	C12—C13—H13A	109.5
C1—C2—H2	108.2	C12—C13—H13B	109.5
C3—C2—H2	108.2	H13A—C13—H13B	109.5
C5—C3—C4	112.2 (3)	C12—C13—H13C	109.5
C5—C3—C2	112.7 (2)	H13A—C13—H13C	109.5
C4—C3—C2	112.0 (2)	H13B—C13—H13C	109.5
C5—C3—H3	106.5	C15—C14—C12	112.5 (2)
C4—C3—H3	106.5	C15—C14—H14A	109.1
C2—C3—H3	106.5	C12—C14—H14A	109.1
C3—C4—H4A	109.5	C15—C14—H14B	109.1
C3—C4—H4B	109.5	C12—C14—H14B	109.1
H4A—C4—H4B	109.5	H14A—C14—H14B	107.8
C3—C4—H4C	109.5	C14—C15—C16	112.0 (2)
H4A—C4—H4C	109.5	C14—C15—H15A	109.2
H4B—C4—H4C	109.5	C16—C15—H15A	109.2
C3—C5—H5A	109.5	C14—C15—H15B	109.2
C3—C5—H5B	109.5	C16—C15—H15B	109.2
H5A—C5—H5B	109.5	H15A—C15—H15B	107.9
C3—C5—H5C	109.5	C10—C16—C15	108.4 (2)
H5A—C5—H5C	109.5	C10—C16—C17	113.7 (2)
H5B—C5—H5C	109.5	C15—C16—C17	114.7 (2)
N1—C6—C7	133.7 (2)	C10—C16—H16	106.5
N1—C6—C9	119.0 (2)	C15—C16—H16	106.5
C7—C6—C9	107.34 (18)	C17—C16—H16	106.5
C6—C7—C8	109.7 (2)	C19—C17—C18	111.2 (3)
C6—C7—Cl1	130.86 (17)	C19—C17—C16	114.0 (2)
C8—C7—Cl1	119.30 (18)	C18—C17—C16	110.9 (3)
O3—C8—O4	121.3 (2)	C19—C17—H17	106.8
O3—C8—C7	129.0 (2)	C18—C17—H17	106.8
O4—C8—C7	109.6 (2)	C16—C17—H17	106.8
O5—C9—O4	110.51 (17)	C17—C18—H18A	109.5
O5—C9—C6	108.15 (19)	C17—C18—H18B	109.5
O4—C9—C6	104.14 (18)	H18A—C18—H18B	109.5
O5—C9—H9	111.3	C17—C18—H18C	109.5
O4—C9—H9	111.3	H18A—C18—H18C	109.5
C6—C9—H9	111.3	H18B—C18—H18C	109.5
O5—C10—C16	106.35 (18)	C17—C19—H19A	109.5
O5—C10—C11	111.29 (19)	C17—C19—H19B	109.5
C16—C10—C11	111.45 (19)	H19A—C19—H19B	109.5
O5—C10—H10	109.2	C17—C19—H19C	109.5
C16—C10—H10	109.2	H19A—C19—H19C	109.5
C11—C10—H10	109.2	H19B—C19—H19C	109.5
C10—C11—C12	111.4 (2)	C6—N1—C2	124.25 (19)
C10—C11—H11A	109.4	C6—N1—H1	117.9
C12—C11—H11A	109.4	C2—N1—H1	117.9
C10—C11—H11B	109.4	C1—O1—H1A	109.5
C12—C11—H11B	109.4	C8—O4—C9	109.00 (16)
H11A—C11—H11B	108.0	C9—O5—C10	116.73 (18)
			
O2—C1—C2—N1	−18.5 (4)	C13—C12—C14—C15	−177.1 (3)
O1—C1—C2—N1	163.8 (2)	C12—C14—C15—C16	56.1 (3)
O2—C1—C2—C3	105.0 (3)	O5—C10—C16—C15	179.41 (19)
O1—C1—C2—C3	−72.8 (3)	C11—C10—C16—C15	58.0 (3)
N1—C2—C3—C5	76.6 (3)	O5—C10—C16—C17	−51.8 (3)
C1—C2—C3—C5	−45.8 (3)	C11—C10—C16—C17	−173.2 (2)
N1—C2—C3—C4	−155.9 (2)	C14—C15—C16—C10	−56.8 (3)
C1—C2—C3—C4	81.8 (3)	C14—C15—C16—C17	175.0 (2)
N1—C6—C7—C8	177.3 (3)	C10—C16—C17—C19	−65.7 (3)
C9—C6—C7—C8	−4.1 (3)	C15—C16—C17—C19	59.8 (3)
N1—C6—C7—Cl1	1.9 (4)	C10—C16—C17—C18	167.9 (3)
C9—C6—C7—Cl1	−179.5 (2)	C15—C16—C17—C18	−66.5 (4)
C6—C7—C8—O3	−175.2 (3)	C7—C6—N1—C2	14.1 (4)
Cl1—C7—C8—O3	0.9 (4)	C9—C6—N1—C2	−164.4 (2)
C6—C7—C8—O4	1.9 (3)	C1—C2—N1—C6	−155.7 (2)
Cl1—C7—C8—O4	177.94 (17)	C3—C2—N1—C6	80.5 (3)
N1—C6—C9—O5	66.0 (3)	O3—C8—O4—C9	178.6 (2)
C7—C6—C9—O5	−112.9 (2)	C7—C8—O4—C9	1.3 (3)
N1—C6—C9—O4	−176.5 (2)	O5—C9—O4—C8	112.3 (2)
C7—C6—C9—O4	4.7 (3)	C6—C9—O4—C8	−3.6 (2)
O5—C10—C11—C12	−176.8 (2)	O4—C9—O5—C10	92.0 (2)
C16—C10—C11—C12	−58.3 (3)	C6—C9—O5—C10	−154.55 (17)
C10—C11—C12—C14	54.4 (3)	C16—C10—O5—C9	168.55 (17)
C10—C11—C12—C13	178.4 (2)	C11—C10—O5—C9	−69.9 (2)
C11—C12—C14—C15	−53.6 (3)		

### Hydrogen-bond geometry (Å, °)

**Table:**

*D*—H···*A*	*D*—H	H···*A*	*D*···*A*	*D*—H···*A*
N1—H1···O2^i^	0.86	2.25	3.019 (3)	148
O1—H1A···O3^ii^	0.82	1.83	2.617 (2)	160

Symmetry codes: (i) *y*+1, *x*−1, −*z*; (ii) −*y*+1/2, *x*−1/2, *z*−1/4.
